# Prevalence of prediabetes and diabetes vary by ethnicity among U.S. Asian adults at healthy weight, overweight, and obesity ranges: an electronic health record study

**DOI:** 10.1186/s12889-022-14362-8

**Published:** 2022-10-22

**Authors:** William S. Vicks, Joan C. Lo, Lynn Guo, Jamal S. Rana, Sherry Zhang, Nirmala D. Ramalingam, Nancy P. Gordon

**Affiliations:** 1 Department of Medicine, Kaiser Permanente Oakland Medical Center, Oakland, CA USA; 2 Division of Research, Kaiser Permanente Northern California, 2000 Broadway, Oakland, CA 94612 USA; 3 The Permanente Medical Group, Oakland, CA USA; 4 Albany Medical College, Albany, NY USA; 5 Department of Cardiology, Kaiser Permanente East Bay, Oakland, CA USA; 6 Graduate Medical Education, Kaiser Permanente Oakland Medical Center, Oakland, CA USA

**Keywords:** Prediabetes, Diabetes, Weight, Obesity, Asian, Chinese, Filipino, South Asian

## Abstract

**Background:**

Asian adults develop Type 2 diabetes at a lower body mass index (BMI) compared to other racial/ethnic groups. We examined the variation in prevalence of prediabetes and diabetes among Asian ethnic groups within weight strata by comparing middle-aged Chinese, Filipino, South Asian, and White adults receiving care in the same integrated healthcare delivery system.

**Methods:**

Our retrospective cross-sectional U.S. study examined data from 283,110 (non-Hispanic) White, 33,263 Chinese, 38,766 Filipino, and 17,959 South Asian adults aged 45–64 years who were members of a Northern California health plan in 2016 and had measured height and weight. Prediabetes and diabetes were classified based on laboratory data, clinical diagnoses, or diabetes pharmacotherapy. Age-standardized prevalence of prediabetes and diabetes were compared by race/ethnicity within healthy weight, overweight, and obesity categories, using standard BMI thresholds for White adults (18.5 to < 25, 25 to < 30, ≥ 30 kg/m^2^) and lower BMI thresholds for Asian adults (18.5 to < 23, 23 to < 27.5, ≥ 27.5 kg/m^2^). Prevalence ratios (PRs) were used to compare the prevalence of diabetes and prediabetes for Asian groups to White adults in each weight category, adjusted for age and BMI.

**Results:**

Across all weight categories, diabetes prevalence was higher for Asian than White adults, and among Asian groups it was highest for Filipino and South Asian adults. Compared to White, PRs for South Asian men/women at healthy BMI were 1.8/2.8 for prediabetes and 5.9/8.0 for diabetes, respectively. The PRs for Filipino men/women at healthy BMI were 1.8/2.6 for prediabetes and 5.0/7.5 for diabetes, respectively. For Chinese men/women at healthy BMI, the PRs for prediabetes (2.1/2.9) were similar to Filipino and South Asian, but the PRs for diabetes were lower (2.1/3.4).

**Conclusion:**

Chinese, Filipino, and South Asian adults have higher prevalence of prediabetes and diabetes than White adults in all weight categories, despite using lower BMI thresholds for weight classification in Asian groups. Within Asian ethnic groups, Filipino and South Asian adults had considerably higher diabetes prevalence than Chinese adults. Our data emphasize the disproportionate metabolic risk among middle-aged Asian adults and underscore the need for diabetes screening among high-risk Asian groups at healthy BMI levels.

## Introduction

The prevalence of diabetes in the US adult population has been steadily rising [1], leading to increased healthcare burden and cost for society and increased morbidity and mortality for affected individuals [2, 3]. The Centers for Disease Control and Prevention [4] (CDC) estimated that in 2019, approximately 14.7% (over 37 million) of U.S. adults had diabetes, and an additional 38% (96 million) of adults had prediabetes, a precursor state of elevated blood glucose levels that frequently progresses to Type 2 diabetes if not managed [3, 5]. Currently, there is limited information about the prevalence of diabetes and prediabetes among Asian American adults, the fastest growing racial/ethnic group in the U.S. [6], comprising approximately 15.4 million adults in 2020 [7]. The CDC estimates that in 2019, 16.7% of Asian American adults aged 18 and older had diabetes and 37% had prediabetes, percentages similar to estimates for non-Hispanic White (White), Black and Hispanic adults [4]. The Patient Outcomes Research To Advance Learning (PORTAL) Network cohort study reported similar estimates for Asian American adults aged ≥ 20 years of 19.3% and 37.1%, respectively, based on 2012–2013 electronic health record (EHR) data for a very large multi-site population of insured adults, with significantly higher prevalence among men than women [8].

Recent research suggests that failure to disaggregate data for individual Asian American ethnic groups can mask meaningful differences in prevalence of health risks and chronic disease [9–20]. The limited studies available suggest that among the three largest U.S. Asian groups, ethnic South Asian and Filipino adults have nearly twice the prevalence of diabetes compared to ethnic Chinese adults (or East Asian), who in some studies have slightly lower or slightly higher diabetes prevalence than White adults [9–11, 13, 16, 18]. Using 2015–2016 EHR data from adults aged 45–85 years in a large Northern California health plan, our group also found significant differences in prevalence of diabetes among Asian ethnic groups, including 31.9% for Filipino, 29.1% for South Asian, and 15.6% for Chinese compared to 12.8% of White adults, with significantly higher diabetes prevalence among men than women [9]. Contemporary estimates of prediabetes prevalence for different Asian ethnic groups in clinical settings remain lacking.

BMI is an independent risk factor for diabetes, but there is growing evidence that Asian Americans develop diabetes at lower BMI levels than other racial/ethnic groups [8, 20–22]. The World Health Organization recommends using lower BMI intervention thresholds for Asians to identify those with a weight-related risk for cardiometabolic disorders [23, 24]. The U.S. Preventive Services Task Force (USPSTF) and the American Diabetes Association (ADA) also recommend screening Asian Americans for diabetes and prediabetes based on a BMI ≥ 23 kg/m^2^, instead of a BMI ≥ 25 kg/m^2 ^[25, 26]. Using standard weight categories for White adults and lower BMI thresholds for Asian adults, the PORTAL Network cohort study showed that Asian adults had an alarmingly higher prevalence of diabetes (10.1%) and prediabetes (33.0%) in the healthy weight category, with prevalence significantly higher among men than women [8]. Another study examined 2011–2018 data from the National Health and Nutrition Examination Survey (NHANES) and found a high prevalence of diabetes and diabetes/prediabetes among Asian American adults aged 35–70 years with healthy weight BMI using standard criteria (BMI 18.5 to < 25 kg/m^2^), including 10.7–12.7% with diabetes among those with BMI in the 21–22 kg/m^2^range [27].

Research has shown that Filipino and South Asian adults have a higher prevalence of overweight and obesity than Chinese adults [20, 24, 28, 29]. Ethnic variation in BMI may in part explain the higher prevalence of diabetes among Filipino and South Asian adults compared to Chinese adults, and comparisons of these ethnic groups to each other or to White adults have generally used models that adjusted for BMI. Fewer studies have examined Asian ethnic group differences in diabetes and prediabetes within weight strata, and the majority of these studies have been based on self-reported data from population surveys. For this study, we used EHR data from an integrated healthcare delivery system in Northern California to compare diabetes and prediabetes prevalence among middle-aged Chinese, Filipino, and South Asian adults within and across healthy weight, overweight, and obesity categories, using White adults for additional comparison. Our aim was to provide data from a clinical setting that could inform efforts to optimize diabetes screening and population management of Asian subgroups at higher risk for developing prediabetes and diabetes, even at a healthy BMI.

## Methods

### Study population

Kaiser Permanente Northern California (KPNC) is an integrated healthcare delivery system that provides care to over 3 million adult members residing in the San Francisco Bay area, Greater Bay Area, Sacramento area, Silicon Valley, and Central Valley. For this cross-sectional study, we used EHR data for White, Chinese, Filipino, and South Asian men and women aged 45–64 years who were KPNC health plan members in 2016 and had measured height and weight. These adults comprise a subset of a previously reported adult cohort [9]. A description of the methods used to assign ethnicity based on race/ethnicity information in the EHR, race/ethnicity data from member surveys, spoken and written language preferences, and surname assignment can be found in the appendix of an earlier 2019 publication in this journal [9]. For analyses comparing the prevalence of prediabetes and diabetes across racial/ethnic groups reported in this study, we used a study sample of 373,098 adults, including 283,110 White, 33,263 Chinese, 38,766 Filipino, and 17,959 South Asian adults who had a BMI ≥ 18.5 kg/m^2^ (i.e., not in the underweight range). The analyses pertaining to prediabetes were restricted to the 320,521 adults who had not progressed to diabetes by the end of 2016; the subset included 250,786 White, 28,829 Chinese, 27,678 Filipino, and 13,228 South Asian adults.

### Study variables

Diabetes status was assigned based on identification by the KPNC’s Diabetes Registry on or prior to December 31, 2016. Members are entered into the Diabetes Registry based on inpatient or outpatient diabetes diagnoses, medications, and/or laboratory criteria [11]. Adults with hemoglobin A1c (HbA1c) ≥ 6.5% or fasting glucose of  ≥ 126 mg/dL in 2016 not yet included in the KPNC Diabetes Registry were also classified as having diabetes in this study. Among those not classified as having diabetes, prediabetes status was assigned based on having ≥ 1 outpatient prediabetes diagnosis in 2015–2016, prediabetes diagnosis in the December 2016 problem list, or having ≥ 1 HbA1c value in the 5.7–6.4% range or ≥ 1 fasting glucose value in the 100–125 mg/dL range in 2016.

Valid ambulatory weight in 2016 and height closest to the date of weight measurement were used to calculate BMI. For White adults, BMI was classified according to standard criteria for healthy weight (BMI 18.5 to < 25.0 kg/m^2^), overweight (BMI 25.0 to < 30.0 kg/m^2^), and obesity (BMI ≥ 30.0 kg/m^2^). For Chinese, Filipino and South Asian adults, we used the lower BMI intervention thresholds recommended for Asians by both the World Health Organization Expert Consultation and the ADA to classify healthy weight (BMI 18.5 to < 23.0 kg/m^2^), overweight (BMI 23.0 to < 27.5 kg/m^2^), and obesity (BMI ≥ 27.5 kg/m^2^) [30, 31].

### Statistical analyses

All statistical analyses were performed using SAS v9.4 (SAS Institute, Cary, NC). We used the cross-tabulation procedure to describe and compare the age group, weight category, and prediabetes and diabetes status distributions of men and women in the four racial/ethnic groups. To facilitate comparisons by race/ethnicity within and across weight categories, we age-standardized the estimates of prediabetes and diabetes prevalence to the 2016 U.S. population aged 45–64 years using the following weights (0.508 for age 45–54 years and 0.492 for age 55–64 years). For the overall study sample and for the subset without diabetes, we used modified log-Poisson regression to determine the prevalence ratio of diabetes and prediabetes, respectively, comparing each Asian ethnic population to the White population within the three weight categories, after additionally adjusting for age and BMI as continuous variables.

## Results

Our study cohort of 171,802 and 201,296 men and women included 132,383 and 150,727 White, 14,170 and 19,093 Chinese, 15,953 and 22,813 Filipino, and 9296 and 8663 South Asian adults, respectively. Table 1 shows the distribution of age group, weight category, and prediabetes or diabetes status by racial/ethnic group. Among men and women, slightly higher percentages of White adults (55–56%) and lower percentages of South Asian adults (39–42%) were in the older age group (55–64 years) compared to Filipino (50–51%) and Chinese (50–54%) adults. Differences in weight category were also seen, especially by sex. Using ethnic-specific BMI thresholds, lower percentages of Filipino (men 7.7%, women 19.7%) and South Asian (men 11.5%, women 17.7%) adults had weight in the healthy range compared to Chinese (men 21.3%, women 46.4%) and White (men 17.1%, women 33.1%) adults. Furthermore, Chinese (men 25.7%, women 14.8%) adults had substantially lower percentages in the obesity range compared to White (men 40.8%, women 36.4%), Filipino (men 49.0%, women 37.5%), and South Asian (men 41.2%, women 41.2%) adults. Adults in all three Asian groups had substantially higher percentages with prediabetes (29–38%) than White (18–25%) women and men. While the sex-specific percentages of Chinese, Filipino, and South Asian adults classified as having prediabetes were similar (lower in women, higher in men, Table 1), the percentages of Filipino (25–34%) and South Asian (21–32%) women and men classified as having diabetes were much higher than the percentages of Chinese (10–17%) adults, and all three Asian groups had higher percentages with diabetes compared to White women and men (9–14%).

**Table 1:** Characteristics of men and women aged 45–64 years in the study cohort

	**White**	**Chinese**	**Filipino**	**South Asian**
**Men**	*N* = 132,383	*N* = 14,170	*N* = 15,953	*N* = 9,296
Age Group				
45–54 years	57,659 (43.6%)	6,562 (46.3%)	7,786 (48.8%)	5,442 (58.5%)
55–64 years	74,724 (56.4%)^a,b,c^	7,608 (53.7%)^b,c,d^	8,167 (51.2%)^a,c,d^	3,854 (41.5%)^a,b,d^
Weight Category^e^				
Healthy weight	22,643 (17.1%)^a,b,c^	3,018 (21.3%)^b,c,d^	1,235 (7.7%)^a,c,d^	1,075 (11.5%)^a,b,d^
Overweight	55,785 (42.1%)^a,c^	7,504 (53.0%)^b,c,d^	6,902 (43.3%)^a,c^	4,395 (47.3%)^a,b,d^
Obesity	53,955 (40.8%)^a,b^	3,648 (25.7%)^b,c,d^	7,816 (49.0%)^a,c,d^	3,826 (41.2%)^a,b^
Prediabetes or Diabetes^f^				
Neither	81,049 (61.2%)^a,b,c^	6,340 (44.7%)^b,c,d^	5,244 (32.9%)^a,c,d^	3,309 (35.6%)^b,c,d^
Prediabetes	32,464 (24.5%)^a,b,c^	5,350 (37.8%)^b,c,d^	5,339 (33.5%)^a,d^	3,043 (32.7%)^a,d^
Diabetes	18,870 (14.3%)^a,b,c^	2,480 (17.5%)^b,c,d^	5,370 (33.7%)^a,d^	2,944 (31.7%)^a,d^
**Women**	*N* = 150,727	*N* = 19,093	*N* = 22,813	*N* = 8,663
Age Group				
45–54 years	66,944 (44.4%)	9,633 (50.5%)	11,455 (50.2%)	5,259 (60.7%)
55–64 years	83,793 (55.6%)^a,b,c^	9,460 (49.5%)^c,d^	11,358 (49.8%)^c,d^	3,404 (39.3%)^a,b,d^
Weight Category^e^				
Healthy weight	49,872 (33.1%)^a,b,c^	8,848 (46.4%)^b,c,d^	4,490 (19.7%)^a,c,d^	1,532 (17.7%)^a,b,d^
Overweight	45,948 (30.5%)^a,b,c^	7,412 (38.8%)^b,c,d^	9,764 (42.8%)^a,d^	3,560 (41.1%)^a,d^
Obesity	54,907 (36.4%)^a,c^	2,833 (14.8%)^b,c,d^	8,559 (37.5%)^a,c^	3,571 (41.2%)^a,b,d^
Prediabetes or Diabetes^f^				
Neither	110,123 (73.1)^a,b,c^	11,261 (59.0%)^b,c,d^	10,451 (45.8%)^a,c,d^	4,328 (50.0%)^a,b,d^
Prediabetes	27,178 (18.0%)^a,b,c^	5,878 (30.8%)^b,d^	6,645 (29.1%)^a,d^	2,548 (29.4%)^d^
Diabetes	13,426 (8.9%)^a,b,c^	1,954 (10.2%)^b,c,d^	5,717 (25.1%)^a,c,d^	1,787 (20.6%)^a,b,d^

^a^Significantly different (*p* < .001) from Chinese

^b^Significantly different (*p* < .001) from Filipino

^c^Significantly different (*p* < .001) from South Asian

^d^Significantly different (*p* < .001) from White

^e^Standard body mass index (BMI, kg/m^2^) categories were used for (non-Hispanic) White adults (healthy weight 18.5 to < 25.0, overweight 25.0 to < 30.0, obesity ≥ 30.0). Asian-specific BMI categories were used for Chinese, Filipino, and South Asian adults (healthy weight 18.5 to < 23.0, overweight 23.0 to < 27.5, obesity ≥ 27.5)

^f^Because the study cohort excluded adults with missing BMI or underweight BMI (< 18.5), the percentages in each weight category with prediabetes or diabetes should not be interpreted as prevalence statistics

**Fig. 1:**
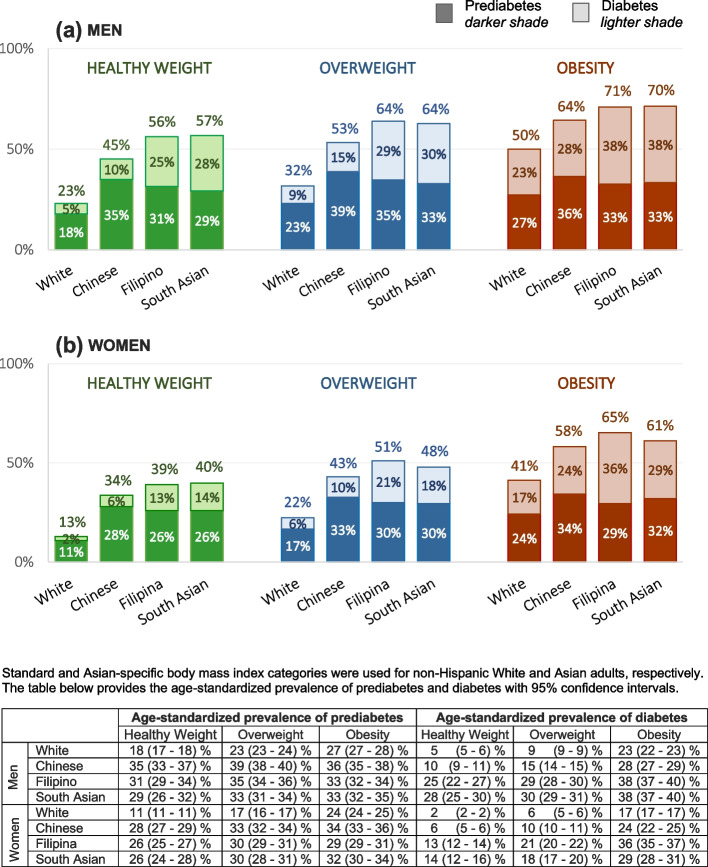
Age-standardized prevalence of diabetes and prediabetes by race/ethnicity and weight category in men and women

Figure 1 shows the age-standardized prevalence of diabetes and prediabetes by race/ethnicity and weight category in men and women. For prediabetes, the prevalence among the three Asian groups (across all weight categories) was much higher than the prevalence of prediabetes among White adults. By healthy weight, overweight, and obesity (ethnic-specific) classifications, respectively, the prevalence of prediabetes was 18%, 23%, and 27% for White men and 29–35%, 33–39%, and 33–36% for men in the Asian groups. Among women, the prevalence of prediabetes by respective weight category was 11%, 17%, and 24% for White women and 26–28%, 30–33%, and 29–34% for women in the Asian groups. Note that the population denominators used for prediabetes estimates include adults identified with diabetes.

The age-standardized prevalence of diabetes by these same respective weight categories (healthy weight, overweight, and obesity) was also substantially lower among White men (5%, 9% and 23%) compared to Chinese men (10%, 15%, and 28%) and Filipino and South Asian men (25–28%, 29–30%, and 38%). Somewhat similar patterns were observed among women for these same respective weight categories, with prevalence lower for White women (2%, 6%, and 17%) compared to Chinese (6%, 10%, and 24%), Filipina (13%, 21%, and 36%) and South Asian (14%, 18% and 29%) women. Overall, the prevalence with diabetes increased with higher weight category. However, the burden of prediabetes was already high among Asian adults in the healthy weight category and was much higher than that observed for healthy weight White adults.

The age-standardized combined prevalence of diabetes and prediabetes within these same weight categories shows how the overall high burden of altered glucose homeostasis differs for these racial/ethnic groups within healthy weight and overweight ranges. In the healthy weight range, about 56% of Filipino and South Asian, 45% of Chinese, and 23% of White men had either diabetes or prediabetes; in the overweight range, this increased to approximately 64% of Filipino and South Asian, 53% of Chinese, and 32% of White men. A similar pattern in the magnitude of differences in prevalence of prediabetes/diabetes by race/ethnicity was seen in women, although the combined prevalence among women was roughly 10 percentage points lower than among men at all weight ranges.

**Fig. 2:**
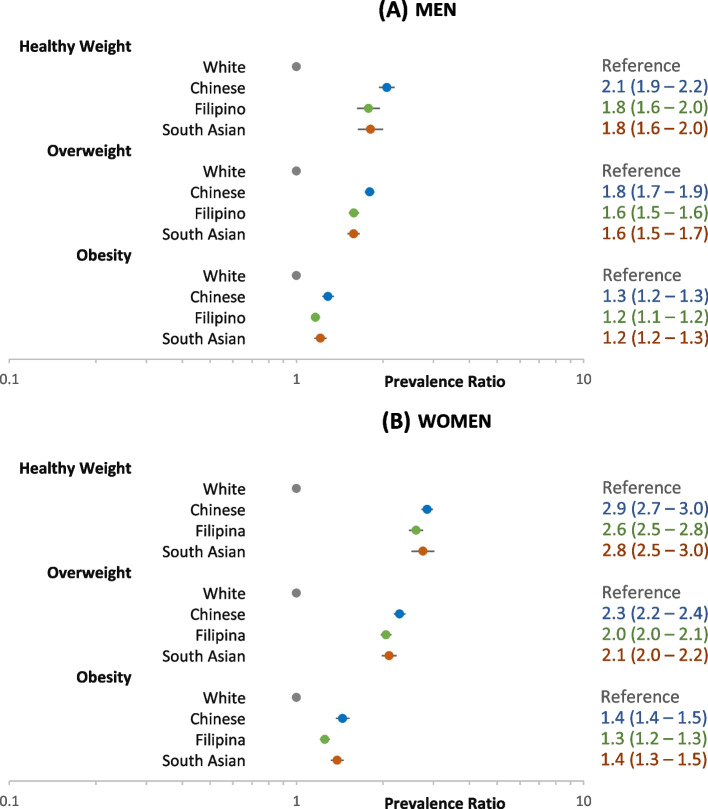
Adjusted prevalence ratio of prediabetes among non-diabetic adults aged 45–64 years

Figure 2 shows the prevalence ratio (PR) of prediabetes for Chinese, Filipino, and South Asian compared to White men and women within each weight category, adjusting for age and BMI. Within each weight category, Chinese, Filipino, and South Asian adults had higher PRs, especially in the healthy weight (Asian-White PRs 1.8–2.1 for men and 2.6–2.9 for women) and overweight categories (Asian-White PRs 1.6–1.8 for men and 2.0–2.3 for women).

**Fig. 3:**
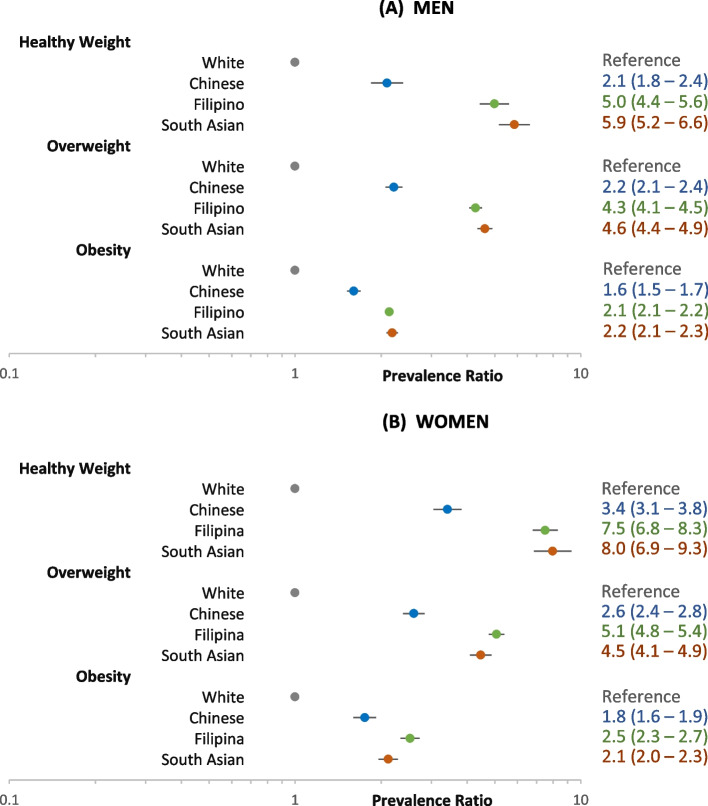
Adjusted prevalence ratio of diabetes among adults aged 45–64 years

Figure 3 shows the age- and BMI-adjusted PR of diabetes for these same groups. The Asian-White PRs for diabetes were also higher in the healthy weight and overweight categories. We further observed much higher PRs for Filipino and South Asian adults, respectively, in the healthy weight (PRs 5.0 and 5.9 for men and 7.5 and 8.0 for women) and overweight (PRs 4.3 and 4.6 for men and 5.1 and 4.5 for women) categories than were seen for Chinese adults in the healthy weight (PR 2.1 for men and PR 3.4 for women) and overweight category (PR 2.2 for men and 2.6 for women).

**Fig. 4:**
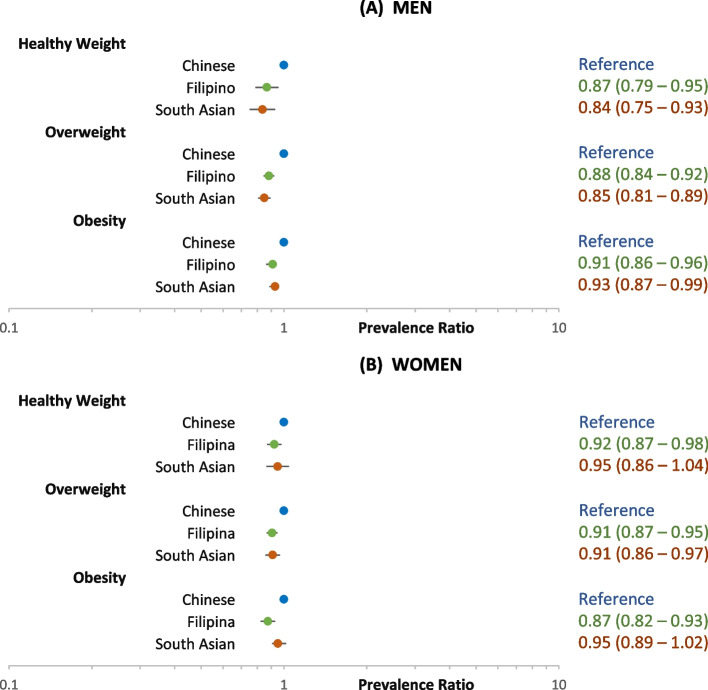
Adjusted prevalence ratio of prediabetes among non-diabetic Asian adults aged 45–64 years

**Fig. 5:**
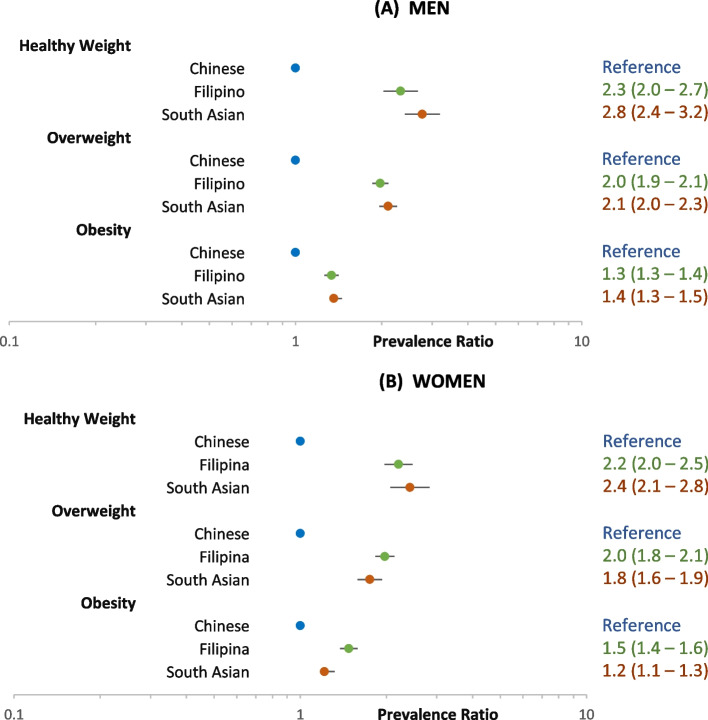
Adjusted prevalence ratio of diabetes among Asian adults aged 45–64 years

Figures 4 and 5 show the adjusted PRs for prediabetes and diabetes, respectively, among the three Asian ethnic groups, using Chinese as the reference. Among men and women, the PR for prediabetes for Filipinos and South Asians compared to Chinese was slightly lower or not significantly different across all weight categories (Fig. 4). However, for diabetes, the PRs for both Filipinos and South Asians compared to Chinese were nearly or more than two-fold higher in the healthy weight and overweight categories, in contrast to smaller differences in the obesity category (Fig. 5).

## Discussion

In this study, we highlight significant differences in diabetes risk and prevalence among specific Asian American populations by BMI level and provide comparisons to White adults from the same integrated healthcare delivery system. Importantly, we observed a substantially higher prevalence of prediabetes and diabetes among Chinese, Filipino, and South Asian adults than White adults within healthy weight, overweight, and obesity BMI levels, despite the use of lower BMI cut-points for Asian adults. The Asian-White differences were greatest at healthy weight BMI levels, with threefold differences in the combined prevalence of diabetes and prediabetes among Asian women and twofold differences among Asian men compared to White counterparts. Filipino and South Asian adults also had much higher prevalence of diabetes compared to Chinese adults, with differences largest among subsets with healthy weight and overweight. Our data underscore the variation at which these populations develop diabetes despite having similar prediabetes burden stratified by BMI level.

Our study results are consistent with previous research documenting significantly higher prevalence of diabetes at lower BMI levels among U.S. South Asian, Filipino, and Chinese adults compared to White adults [20, 24, 32–35]. In addition, a key finding of our study is the differential burden of diabetes risk among specific Asian American populations that was most notable at lower BMI levels; the largest differences in diabetes prevalence relative to White adults were observed among South Asian and Filipino adults in the healthy BMI range, both higher than for Chinese adults. In contrast, the prediabetes prevalence in these two Asian groups compared to White adults were within range of those observed for Chinese compared to White adults at healthy BMI levels. These findings emphasize the heterogeneity in glucose dysmetabolism among Asian subgroups and point to the need for further examination of contributing factors, including diet and lifestyle, genetic predisposition, body composition, and metabolic differences between groups that may contribute to increased diabetes risk and the variable rate at which these groups develop diabetes.

Studies conducted in Asia and Europe have documented a dramatic increase in Type 2 diabetes in non-U.S. Asian populations. Over the past two decades, the prevalence of prediabetes and diabetes has increased in both China [36] and the Indian subcontinent [37, 38]. In the United Kingdom, the prevalence of prediabetes was observed to be higher in Chinese and even higher in South Asian adults compared to White adults [39]. This rising trend is reflected in the U.S. where the prevalence of diabetes and prediabetes in South Asian immigrants have been reported to be as high 17.4% and 33%, respectively [40]. Another study of immigrants in the U.S. found that those from the Indian subcontinent and Southeast Asia had higher age-adjusted prevalence of diabetes than those from Europe [41]. A 9-year follow up study of a cohort of Filipino adults found an estimated 16.3% incidence rate of type 2 diabetes between 1998 and 2007 [42].

Currently, the ADA and USPSTF recommend diabetes screening for Asian Americans with a BMI ≥ 23 kg/m^2 ^beginning at age 35 years [25, 26]. However, based on their analyses of NHANES data, Aggarwal, et. al., recommend initiating diabetes and prediabetes screening for Asian Americans at an even lower BMI of 20 kg/m^2^ or greater, suggesting that this would be equivalent to screening White adults with a BMI ≥ 25 kg/m^2 ^[27]. The American Association of Clinical Endocrinology further recognizes the risk of diabetes among adults with normal range BMI and recommends against using BMI as an indicator for initiating screening for diabetes, referencing multiple large cohort studies that report 9–21% of adults diagnosed with diabetes had BMIs in the normal (healthy) weight range [43]. Our data also call for greater attention to screening of diabetes and prediabetes in Asian American adults in the healthy weight range, especially those of Filipino and South Asian ethnicity.

Our study has several strengths. First, these results are based on comprehensive EHR data for a very large representative cohort of health plan members that received care from the same integrated healthcare delivery system. Most previous studies of variation in diabetes prevalence across Asian ethnic groups have been based on self-reported data regarding diabetes status, height, and weight, in the absence of anthropometry, laboratory data, and clinical diagnoses. We were able to categorize adults as having diabetes or prediabetes based on a combination of ICD codes, laboratory findings, and pharmacy records, and to use in-clinic height and weight measurements to calculate BMIs. Second, the large size of our Asian ethnic groups enabled us to focus our analyses on middle-aged adults rather than the full adult age spectrum and to conduct separate comparisons of prediabetes and diabetes prevalence by sex and Asian ethnicity within the three weight categories. We were further able to use EHR data for a single 2016 population-based study cohort, in contrast to many previous studies that have required pooling of data across multiple survey cycles to obtain enough adults in different Asian ethnic groups for similar analyses. Third, our study subgroups included both English and non-English speakers, the latter often being under-represented in population-based surveillance surveys.

We acknowledge that our study has some limitations. First, we report findings from a clinical population not systematically screened at the same time, contributing to under-ascertainment of prediabetes and diabetes. However, ethnic trends in prediabetes prevalence were similar when restricting prediabetes analyses to those with laboratory testing (data not shown) and our study focused on adults aged ≥ 45 years (the age threshold at which universal screening for diabetes was recommended by the ADA prior to 2022) [44] to minimize the potential influence of preferential diabetes screening among younger Asians. Second, while we were able to use clinic-measured BMI, we did not have information about distribution of adiposity, including central adiposity which may play a greater role in the higher prevalence of diabetes and prediabetes among Asian adults [30, 45, 46]. Third, our cohort excluded adults without a clinic-measured BMI in 2016 and thus may not reflect findings in the broader population of adults that include those who do not come into clinic for routine medical care. Fourth, we acknowledge the limitations in our study with regard to ascertainment of race/ethnicity [9], including data derived from the EHR, and the lack of data to examine acculturation factors, including country of birth, length of residence in the U.S., and culture-related dietary practices that might predispose Asian adults to develop prediabetes/diabetes. Finally, our cohort was drawn from a single health plan in Northern California and may not be generalizable to other U.S. regions or healthcare systems.

## Conclusion

This study highlights the importance of prediabetes and diabetes assessment in Asian Americans and the variation in risk and disease burden among different Asian ethnicities. Among Asian adults with BMI in the healthy range, up to one-third of men and one-fourth of women had prediabetes and more than one-fourth of Filipino and South Asian men had diabetes. South Asian ethnicity is considered a “risk enhancer” for future cardiovascular disease for both men and women [47] and clustered cardiometabolic risk has been reported among Filipino and South Asian adults in this population [48]. Our findings further highlight the disproportionate metabolic risk among different Asian groups across all weight categories and underscore the desirability of broadening prediabetes and diabetes screening recommendations for higher-risk middle-aged Asian subgroups to include screening at healthy BMI levels. We believe that our findings are directly applicable to similar healthcare systems, highlight key population trends, and may inform future prospective studies and efforts to target screening in Asian populations where the burden of diabetes and prediabetes is under-recognized.

## Data Availability

The data supporting the results of this study are not publicly available due to privacy laws associated with medical data. Questions and requests regarding data availability should be addressed to the corresponding author (NPG).

## Abbreviations

ADA: American Diabetes Association
BMI: Body mass index
CI: Confidence interval
EHR: Electronic health record
HbA1c: Hemoglobin A1c
IFG: Impaired fasting glucose
KPNC: Kaiser Permanente Northern California
IGT: Impaired glucose tolerance
NHANES: United States National Health and Nutrition Examination Survey
PR: Prevalence ratio
U.S.: United States
USPSTF: United States Preventative Services Task Force
